# Prevalence and determinants of dental visits among older adults: findings of a nationally representative longitudinal study

**DOI:** 10.1186/s12913-019-4427-0

**Published:** 2019-08-20

**Authors:** K. Spinler, G. Aarabi, R. Valdez, C. Kofahl, G. Heydecke, H.-H. König, A. Hajek

**Affiliations:** 1 Department of Prosthetic Dentistry, Center for Dental and Oral Medicine, University Medical Center Hamburg-Eppendorf, Hamburg, Germany; 2 Institute of Medical Sociology, Center of Psychosocial Medicine, University Medical Center Hamburg-Eppendorf, Hamburg, Germany; 3 Department of Health Economics and Health Services Research, University Medical Center Hamburg-Eppendorf, Hamburg, Germany

**Keywords:** Health care use, Dental services utilization, Dental visits, Longitudinal study, Older adults, German ageing survey

## Abstract

**Background:**

The first aim was to present descriptive data on the frequency of dental visits among older adults in Germany. The second aim was to identify the determinants of the number of dental visits using a longitudinal approach.

**Methods:**

Longitudinal data were derived from the German Ageing Survey, which is a nationally representative sample of community-dwelling individuals ≥40 years in Germany. The frequency of dental visits in the past 12 months was recorded in the years 2002, 2008 and 2011. In order to control for time-constant unobserved heterogeneity, Poisson fixed effects regressions were used.

**Results:**

While the mean number of dental visits was 2.3 (SD: 2.0) in 2002, it was 2.0 (SD: 1.7) in 2008 and 2.1 (SD: 1.7) in 2011. The frequency of dental visits declined with age (total sample and women), transitions from normal weight to overweight (total sample), changes from divorced/widowed/single/married, living separated from spouse to ‘married, living together with spouse’ in women and with a decrease in the number of physical illnesses (total sample and men).

**Conclusions:**

The frequency of dental visits declines with age in older adults. While some of the determinants of frequency are non-modifiable (e.g., ageing and worsening of general health), others are modifiable (e.g., change in weight category).

## Background

Over the past decades, it has become evident that oral health and overall health are intrinsically linked. For instance, tooth loss was found to be associated with an increased risk of ischemic stroke and poor mental health [1–3]. Research also suggests that early detection and treatment of periodontitis have beneficial effects on cardiovascular and metabolic diseases [4–7]. Furthermore, poor oral health has a negative influence on quality of life [1].

The oral health care of older adults is a matter of increasing importance for three reasons: i) the increasing number and proportion of older adults, ii) the increasing life expectancy resulting in a growing number of old-olds, and iii) changes in oral health status of older people towards more remaining teeth until the end of life [8]. These demographic change factors, combined with the increasing complexity of dental treatment of older patients, require a specific focus in dental medical research in order to provide best oral health care for older people [9].

In Germany, population-based dental research is continuously conducted via the German Oral Health Surveys (DMS). The fifth iteration of this survey (DMS V, also the latest survey) responded to the demographic changes and thus gave a higher priority to collecting data on older adults than in the previous surveys [8]. Not surprisingly, the data of DMS V show poorer oral health among older people (65+ years old) compared to younger participants (18–64 years old) [8]. However the data demonstrate a general improvement in oral health, with a significantly lower prevalence of tooth loss and periodontitis in older adults in 2016 (DMS V) than in 1997 (DMS III) [10]. Besides personal oral hygiene and self-care, regular preventive visits to the dentist and – if necessary – dental treatments are essential to promote and maintain good oral health. The latter is supported by the results of the DMS V study, in which patients who visited a dentist for regular check-ups showed a better oral health status [8]. Despite the importance of regular dental visits as well as the growing usage of them, data from previous surveys of the DMS (III & IV) show that older adults are still using dental services less frequently than younger adults [10, 11]. These results are consistent with current observations of one of Germany’s biggest health care insurance providers (the BARMER Ersatzkasse), which show that 71.5% of their insured persons in total visited a dentist at least once in 2016. Older insurees (75+ years old), however, showed a significantly less frequent utilization of dental services, of under 60% [12]. Regular dental check-ups have a preventive effect on oral health, specifically on the development of periodontitis, and are recommended one to two times a year for adults [8, 13–15].

Nitschke et al. showed that dental service utilization is negatively associated with age, although the utilization of other medical services increases with age [16]. The additional influencing factors on dental service utilization included presence of acute pain symptoms, a need for prosthetic treatment, education level, and financial situation. In addition, individuals with no or less functional limitations and better mental health tended to use dental services more frequently. The reverse conclusion would be that disabilities, immobility, or symptoms of depressive disorders or dementia hinder dentist visits among older people.

We are aware of factors influencing dental service utilization, but there is a lack of longitudinal studies investigating the determinants of dental visits. Although the DMS surveys are comprehensive and the largest in Germany in previous decades, only cross-sectional studies have been conducted on this topic so far. With the present study, we are closing this gap by analyzing determinants of dental service utilization of the German Ageing Survey (DEAS) data longitudinally, which offers a better understanding about which life changes influence service utilization over time.

In the current study, longitudinal data from several waves of the DEAS were used i) to present descriptive data on the frequency of dental visits among older adults in Germany, and ii) to identify the determinants of dental service utilization in this older population group.

## Methods

### Sample

For this study, data were derived from the DEAS, which started in 1996 and is funded by the German Federal Ministry for Family Affairs, Senior Citizens, Women, and Youth (BMFSFJ). The survey is a nationwide, representative, combined cross-sectional and longitudinal study of the community-dwelling population ≥ 40 years in Germany, organized by the German Centre of Gerontology (DZA). Among other things, participants are asked about their employment status, health, social support, and well-being.

The sample was drawn by means of national probability sampling (i.e., a systematic random sample of individuals ≥40 years in Germany was selected). To this end, data from compulsory registration in Germany was used. First, 90 eastern and 200 western communities in Germany were selected. Second, a systematic random sample for each community was drawn via interval sampling. The data sets for this study are from the second (2002), third (2008) and fourth wave (2011) of the DEAS-survey. In the second wave, which had a response rate of 38%, 5194 individuals took part. In the third wave, which also had a response rate of 38%, 8200 individuals were interviewed. In the fourth wave (56% response rate), 4855 individuals participated. The varying number of participants can be explained by the fact that the DEAS study has a cohort-sequential design. According to Neller [17], these response rates correspond to response rates of other large surveys conducted in Germany. While new samples were introduced in the second and in the third wave, the fourth wave was just a panel survey (i.e., only individuals who already took part in former waves were included in the fourth wave). In the second wave, 1526 individuals from 1996 were re-interviewed while in the third wave, 6205 new participants were included and 1995 participants of the former waves were re-interviewed. The main reasons for lacking follow-up data were death, poor general health and refusal to participate. Further details are provided elsewhere [18].

### Dependent variable

The number of dental visits in the preceding 12 months was used as outcome measure. In all three waves, attendance was measured as “never”, “once”, “2–3 times”, “4–6 times”, “7–12 times”, or “more often” (open answer). In accordance with Bock et al. [19], attendance was recoded as “never” = 0; “once” = 1; “2–3 times” = 2.5; “4–6 times” = 5; “7–12 times” = 9.5; and “more often” = 13.

### Independent variables

Based on previous studies [16, 20–25] and theoretical considerations, the following explanatory variables were selected: age (in years), employment status (employed; retired; other: not employed) and marital status (married, living together with spouse; other (including: single; widowed; divorced; married, living separated from spouse). Concerning marital status, the rationale for the dichotomization was to use marital status as a proxy for living alone or living with a partner. We also included the self-rated body-mass-index (BMI). BMI thresholds were classified according to the World Health Organization (WHO) parameters: underweight (BMI < 18.5 kg/m^2^), normal weight (18.5 kg/m^2^ ≤ BMI < 25 kg/m^2^), overweight (25 kg/m^2^ ≤ BMI < 30 kg/m^2^), and obese (BMI ≥ 30 kg/m^2^). In addition, we included self-rated health (from 1 = very good to 5 = very bad) and the number of physical illnesses (hearing problems, ear problems; vision impairment, eye problems; bladder problems; liver or kidney problems; gall bladder; diabetes; cancer; stomach and intestinal problems; respiratory problems, asthma, shortness of breath; joint, bone, spinal or back problems; bad circulation; cardiac and circulatory disorders). Furthermore, we included depression (Center for Epidemiological Studies Depression Scale, CES-D ≥ 18 [26] and loneliness. The loneliness scale developed by Gierveld and van Tilburg [27] has good psychometric properties. The scale consists of six items (four levels each: 1 = “strongly agree”, 2 = “agree”, 3 = “disagree” to 4 = “strongly disagree”). It is a short version of the 11-item De Jong Gierveld Loneliness Scale which has very good psychometric properties [28, 29]. By averaging the scores on the six items, the scale was constructed. Higher values reflect higher perceived loneliness scores. Cronbach’s alpha was .83 in our study.

### Statistical analysis

In this study, Poisson fixed effects regressions (FE regressions) were used because this regression model is suited to controlling for time-constant unobserved factors (e.g., genetic disposition) in large survey studies [30]. Fixed effects regressions yield consistent estimates under weak assumptions [31], which was supported by a Hausman test (Chi^2^ = 39.47, *p* < .001) in our study.

FE regressions solely use changes within individuals over time (e.g., changes from employment to retirement within an individual over time; e.g., from year 2002 to year 2008). For this reason, the FE estimator is also called “within estimator”. This is why only time-dependent variables (e.g., marital status, age, or the number of chronic illnesses) can be included in FE regressions as main effects. Time-invariant factors (factors that do not vary within individuals over time, e.g., gender or genetic disposition) cannot be included as main effects in FE regressions. Cluster-robust standard errors were computed [32]. The statistical significance was defined as *p*-value of 0.05 or smaller. Analyses were conducted using Stata 15.1 (StataCorp., College Station, Texas, USA).

## Results

### Sample characteristics

Sample characteristics for individuals included in the Poisson FE regression analysis are described in Table 1. For descriptive purposes, the time-constant variables gender and education (International Standard Classification of Education, ISCED-97 [33], with three categories: low (0–2), medium (3–4), and high (5–6)) are also reported.

**Table 1:** Sample characteristics for individuals included in Poisson FE regression analysis (*n* = 7299 observations – based on 3331 individuals)

	N/Mean	%/(SD)
Gender: Female	3594	49.2%
Education (ISCED-97): - Low	339	6.3%
- Middle	2712	50.7%
- High	2304	43.0%
Age in years	62.7	(10.9)
Marital status: Married, living together with spouse	5578	76.4%
Employment status:		
- Employed	2745	37.6%
- Retired	3684	50.5%
- Other: not employed	870	11.9%
Weight categories:		
- Underweight	27	0.4%
- Normal weight	2782	38.1%
- Overweight	3093	42.4%
- Obesity	1397	19.1%
Number of physical illnesses	2.3	(1.8)
Self-rated health (1 = very good to 5 = very bad)	2.4	(0.8)
Absence of depression (CES-D < 18)	6876	94.2%
Loneliness (1 = low loneliness scores to 4 = high loneliness scores)	1.7	(0.5)
Number of dental visits	2.1	(1.8)

Notes: *N* = number; *SD* = standard deviation. Underweight: BMI < 18.5 kg/m^2^, normal weight: 18.5 kg/m^2^ ≤ BMI < 25 kg/m^2^, overweight: 25 kg/m^2^ ≤ BMI < 30 kg/m^2^), and obese: BMI ≥ 30 kg/m^2^; CES-D = Center for Epidemiological Studies Depression Scale; Loneliness was quantified using the short version of the 11-item De Jong Gierveld Loneliness Scale. ISCED-97 was used to quantify education

Among these individuals, 49.2% were female. Mean age was 62.7 years (SD: 10.9 years, ranging from 40 to 95 years). The mean number of dental visits was 2.1 (SD: 1.8). Further details are displayed in Table 1.

**Fig. 1:**
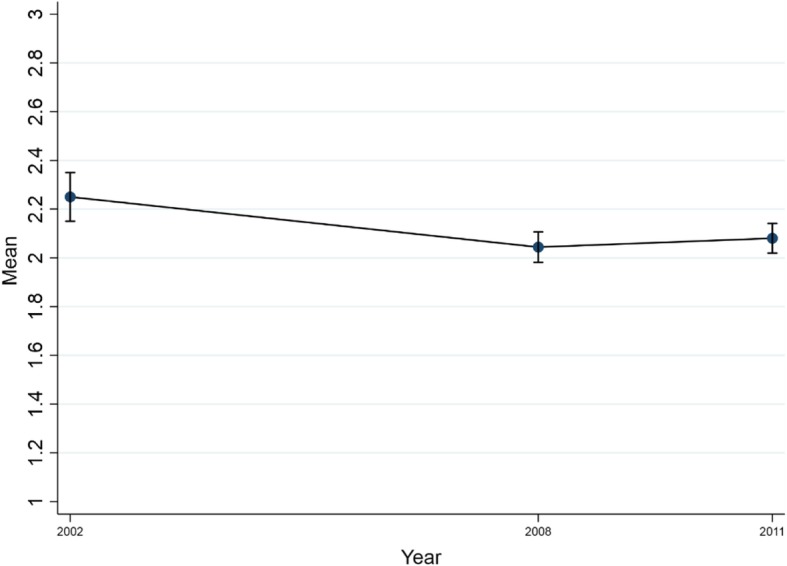
Mean number of dental visits from 2002 to 2011

In 2002 (see Fig. 1), the mean number of dental visits was 2.3 (SD: 2.0), while it was 2.0 (SD: 1.7) in 2008 (*p* < .001) and 2.1 (SD: 1.7) in 2011 (*p* < .01). In comparison, the mean number of general practitioner (GP) visits was 4.3 (SD: 3.7) in 2002, 3.8 (SD: 3.1) in 2008 and 3.8 (SD: 3.0) in 2011.

### Regression analysis

The results of the Poisson FE regression analyses are summarized in Table 2. The frequency of dental visits decreased with age (total sample [β = −.01, *p* < .001], was lower among women [β = −.02, p < .001]), and decreased with transitions from normal weight to overweight (total sample [β = −.09, *p* < .05]). The frequency of dental visits also decreased with changes from divorced/widowed/single/married, living separated from spouse to ‘married, living together with spouse’ (women [β = .28, *p* < .01]), and finally with a decrease in the number of physical illnesses (total sample [β = .03, *p* < .05] and men [β = .03, *p* < .05]). Other explanatory variables were not significantly associated with the outcome measure.

**Table 2:** Determinants of dental visits

	Outcome measure: Frequency of dental visits in the preceding 12 months		
Independent Variables	Total sample	Men	Women
Age	−0.01***	−0.01	− 0.02***
	(0.00)	(0.01)	(0.01)
Marital status (Reference category: Married, living together with spouse): Other (Singles; Widowed; Divorced; Married, living separated from spouse)	0.14+	−0.01	0.28**
	(0.08)	(0.13)	(0.11)
Employment status (Reference category: Employed): - Retired	−0.07	−0.11	− 0.05
	(0.05)	(0.08)	(0.08)
- Other: not employed	−0.03	0.06	−0.08
	(0.05)	(0.08)	(0.07)
Weight categories (Reference category: Normal weight): - Underweight	−0.30	− 0.04	− 0.36
	(0.31)	(0.46)	(0.38)
- Overweight	−0.09*	−0.12+	− 0.06
	(0.05)	(0.07)	(0.06)
- Obesity	−0.08	−0.17	0.01
	(0.08)	(0.11)	(0.11)
Number of physical illnesses	0.03*	0.03*	0.03
	(0.01)	(0.02)	(0.02)
Self-rated health (1 = very good to 5 = very bad)	−0.02	−0.00	− 0.03
	(0.02)	(0.03)	(0.03)
Presence of depression (Reference category: Absence of depression) (CES-D ≥ 18)	−0.08	−0.09	− 0.09
	(0.06)	(0.09)	(0.07)
Loneliness (1 = low loneliness scores to 4 = high loneliness scores)	−0.04	−0.05	− 0.02
	(0.04)	(0.05)	(0.05)
Observations	7299	3705	3594
Individuals	3331	1688	1643

Results of Poisson FE regressions

Beta coefficients were reported; cluster robust standard errors in parentheses; *** *p* < 0.001, ** *p* < 0.01, * *p* < 0.05, + *p* < 0.10; Underweight: BMI < 18.5 kg/m^2^, normal weight: 18.5 kg/m^2^ ≤ BMI < 25 kg/m^2^, overweight: 25 kg/m^2^ ≤ BMI < 30 kg/m^2^), and obese: BMI ≥ 30 kg/m^2^; CES-D = Center for Epidemiological Studies Depression Scale; Loneliness was quantified using the short version of the 11-item De Jong Gierveld Loneliness Scale

## Discussion

The main finding of this study was that the frequency of dental visits declined with increasing age in the total sample and in women, transitions from normal weight to overweight in the total sample, a decrease in the number of physical illnesses in the total sample and in men. Furthermore, the frequency of dental visits increased with changes from ‘married, living together with spouse’ to another marital status (in women only).

While previous cross-sectional studies found that older adults utilize dental services less often than younger adults, our longitudinal study extends this knowledge by finding that the frequency of dental service utilization declines with rising age. Nitschke et al. found these cross-sectional results for Switzerland, the DMS III – V and others for Germany, and Wall and Brown had observed similar utilization among the elderly in the USA [8, 11, 16, 34]. Our finding adds validity to older adults’ higher risk to underutilize dental services compared to younger age groups. Underutilization means, that adults visit the dentist less than one to two times a year [13–15]. The present results show that not only interindividual but also intraindividual explanations are needed to describe dental service utilization in older age.

A further intraindividual change that was observed concerns BMI, whereby those who shifted from normal- to overweight visited the dentist less often. It is known that obesity is a strong risk factor for a number of chronic diseases e.g. cardiovascular diseases, diabetes and chronic back pain [35]. Underlining our results, Holm-Petersen et al. proposed that, with rising number of obesity-related diseases, the patients’ focus shifts to general medical appointments rather than dental visits [23]. A high BMI is additionally associated with unhealthy nutrition, which is linked to diabetes, a higher risk of oral diseases and lower self–care behavior [36–39]. Furthermore, a recent study demonstrated the correlation between self-care/self-efficacy, diabetes and oral health behavior. Low self-care/self-efficacy can negatively influence both oral health behavior and the frequency of dental visits [40]. Although these high-risk patients present an increased need for dental treatment, our data suggests that they visit the dentist less often. It may be assumed, that some patients will not go to the dentist simply because they do not experience pain or other noticeable symptoms [41].

Women in our study used dental services more frequently after experiencing a change in marital status from ‘married, living together with spouse’ to another marital status. Previous research indicated that different marital statuses or changes in marital statuses can have both healing or detrimental effects on general health and influence individuals’ health behavior [42–45]. Published data further show that changes of the marital status can have more influence on an individual’s health and health service use than a stable marital status. The greatest impact can be seen in persons who are newly widowed or newly divorced [43]. However, the direction in which changes in marital status influences health care visits is controversial [46–49], due to the myriad of reasons for changes in marital status. One interpretation of our results is based on the assumption that a significant proportion of widows may have spent a long period of time as the family caregiver for their dependent husbands until their husband’s death. It is well known that family caregiver (mostly women) can be very absorbed and burdened by the care they provide, often for years [50, 51]. Therefore, it is not unlikely that widows become more self-aware and invest more time in self-care after this period. Overall, however, little is known about the influence of changes of the marital status in relation to dental service use. Further research is needed to clarify this.

In accordance with a recent study by Hajek et al., which focuses on health care utilization in general, we observe a correlation between a rising number of illnesses and an increase of dental service utilization [52]. It seems plausible that a rising number of illnesses may lead to an increase of illness-related symptoms. Patients therefore try to counter this need inter alia by more dentist visits.

After analyzing the study results, we assume that insufficient utilization of dental services with rising age is the result of an accumulation of risk factors. Therefore, the combination of changes in age, general health status and weight must be addressed altogether and cannot be considered separately.

### Strengths and limitations

While there are some studies addressing the prevalence of dental visits in Germany, there is a lack of studies investigating the general determinants of dental visits longitudinally using intra-individual variation. This is the first study that has illustrated the prevalence of dental visits among older adults in Germany *and* has identified general determinants of dental visits using a *longitudinal* approach. The problem of time-constant unobserved heterogeneity, which is a main challenge in large survey studies, was reduced by using FE regressions. In the same vein, it is sometimes perceived as a shortcoming of the FE strategy that time-constant factors cannot be included as main effects in the regression model. However, as already argued by Brüderl and Ludwig [30], this is a key strength of the FE estimator because, for example, genetic differences between individuals do not bias the estimates. In large survey studies, it is almost impossible to include this potential confounder. The widely acknowledged nationally representative DEAS study was conducted including well-validated instruments (e.g., CES-D).

Some limitations are worth acknowledging: A small sample selection bias has been observed in the DEAS study. This means that the participation rate tends to be lower, for example, in the oldest old. Oral health-related variables, like e.g. DMF(T) Index (Decayed/Missing/Filled Teeth), PBI (Papillary Bleeding Index) and API (Approximal Plaque Index), are missing in the DEAS study. These would have provided information about the oral health of individuals and given greater insight into their use of routine dental check-ups for example [53]. The frequency of dental visits in the past 12 months was used. Hence, the possibility of a small recall bias cannot be dismissed. However, a 12 months period is in accordance with previous recommendations [54]. We cannot distinguish between therapeutic and preventive dental visits. Therefore, for example, while therapeutic visits might have increased, preventive visits might have decreased (or vice versa). Furthermore, self-rated BMI was used. Although self-reported height and weight data were shown to be valid for identifying relationships in epidemiological studies [55], it cannot be dismissed that some people slightly overestimated height and underestimated weight. Since the Poisson fixed effects regression that was employed in our study uses changes within individuals over time rather than absolute BMI values, major effects on the results resulting from slight inaccuracies of weight and height absolute values are unlikely.

## Conclusion

The frequency of dental visits in older adults is determined by increasing age and several other determinants, some of which are non-modifiable (e.g., ageing) and others that are modifiable (e.g., change in weight category). Aging with all its combined factors and linked changes can be seen as a risk factor for low dental service utilization. The impeding circumstances which are adding on with rising age and which are changing the utilization behavior need to be addressed additionally and specifically. There is a need for greater awareness of older persons’ oral health needs and prevention programs that focus especially on these needs.

## Data Availability

The data used in this study are third-party data. The anonymized data sets of the DEAS (1996, 2002, 2008, 2011, and 2014) are available for secondary analysis. The data has been made available to scientists at universities and research institutes exclusively for scientific purposes. The use of data is subject to written data protection agreements. Microdata of the German Ageing Survey (DEAS) is available free of charge to scientific researchers for non-profitable purposes. The FDZ-DZA provides access and support to scholars interested in using DEAS for their research. However, for reasons of data protection, signing a data distribution contract is required before data can be obtained. Please see for further Information (data distribution contract): https://www.dza.de/en/fdz/german-ageing-survey/access-to-deas-data.html
